# A deep learning-based automated diagnosis system for SPECT myocardial perfusion imaging

**DOI:** 10.1038/s41598-024-64445-2

**Published:** 2024-06-12

**Authors:** Dai Kusumoto, Takumi Akiyama, Masahiro Hashimoto, Yu Iwabuchi, Toshiomi Katsuki, Mai Kimura, Yohei Akiba, Hiromune Sawada, Taku Inohara, Shinsuke Yuasa, Keiichi Fukuda, Masahiro Jinzaki, Masaki Ieda

**Affiliations:** 1 Department of Cardiology, Keio University School of Medicine, 35 Shinanomachi, Shinjuku-Ku, Tokyo, 160-8582 Japan; 2 Center for Preventive Medicine, Keio University School of Medicine, 35 Shinanomachi, Shinjuku-Ku, Tokyo, 160-8582 Japan; 3 Department of Radiology, Keio University School of Medicine, 35 Shinanomachi, Shinjuku-Ku, Tokyo, 160-8582 Japan; 4 Department of Cardiovascular Medicine, Okayama University Hospital, Okayama, 700-8558 Japan

**Keywords:** Cardiology, Cardiovascular diseases, Computer science

## Abstract

Images obtained from single-photon emission computed tomography for myocardial perfusion imaging (MPI SPECT) contain noises and artifacts, making cardiovascular disease diagnosis difficult. We developed a deep learning-based diagnosis support system using MPI SPECT images. Single-center datasets of MPI SPECT images (n = 5443) were obtained and labeled as healthy or coronary artery disease based on diagnosis reports. Three axes of four-dimensional datasets, resting, and stress conditions of three-dimensional reconstruction data, were reconstructed, and an AI model was trained to classify them. The trained convolutional neural network showed high performance [area under the curve (AUC) of the ROC curve: approximately 0.91; area under the recall precision curve: 0.87]. Additionally, using unsupervised learning and the Grad-CAM method, diseased lesions were successfully visualized. The AI-based automated diagnosis system had the highest performance (88%), followed by cardiologists with AI-guided diagnosis (80%) and cardiologists alone (65%). Furthermore, diagnosis time was shorter for AI-guided diagnosis (12 min) than for cardiologists alone (31 min). Our high-quality deep learning-based diagnosis support system may benefit cardiologists by improving diagnostic accuracy and reducing working hours.

## Introduction

Cardiovascular diseases are the leading cause of single organ-associated death in numerous countries, with a rapidly increasing global incidence. Coronary artery disease (CAD), including myocardial infarction and angina, is the main etiology of cardiovascular disease. Single-photon emission computed tomography for myocardial perfusion imaging (MPI SPECT) can visualize cardiac blood flow using radioisotopes and is one of the most common non-invasive examinations for coronary artery disease (CAD) diagnosis^1–4^. MPI SPECT is crucial in predicting clinical outcomes and in making clinical decisions, such as the need for coronary revascularization^5–7^. However, there are unavoidable issues in diagnosing CAD with MPI SPECT, with the primary concern being that images obtained from MPI SPECT sometimes contain various type of noises and artifacts, which reduce the diagnostic accuracy. One example is artifactual attenuation defect in the inferior wall due to diaphragmatic motion, which can be interpreted as a perfusion abnormality^8–15^. Through specialized training for medical doctors, in which imaging characteristics of various artifacts are taught, it is possible to determine whether artifacts are the cause of decreased uptake in MPI SPECT imaging. However, inadequate interpretation can lead to artifacts being diagnosed as CAD, resulting in an increase in false positives^16^. Furthermore, although over 220,000 cardiovascular SPECTs are performed annually in Japan^17^, the number of nuclear medicine specialists is only about 2700^18^. Thus, especially in local hospitals, it is often difficult to interpret MPI SPECT images due to the absence of nuclear medicine specialists.

To overcome this drawback, automated diagnostic systems for MPI SPECT images have been developed using machine learning^19–23^, which have been further augmented using deep learning techniques in recent years^24–28^. Although the performance of machine learning has notably been improved through the utilization of deep learning, certain limitations remain that hinder diagnostic accuracy. First, the input data often consists of two-dimensional (2D) data, such as polar maps^25,28^, or only stress loading images^27^; therefore, portions of information originally possessed by a SPECT image become lost. Second, CAD lesions should be central to the construction of models that enable localization of decreased blood flow. However, they are examined only in a limited number of cases^28^; therefore, the number of training datasets is insufficient.

**Figure 1:**
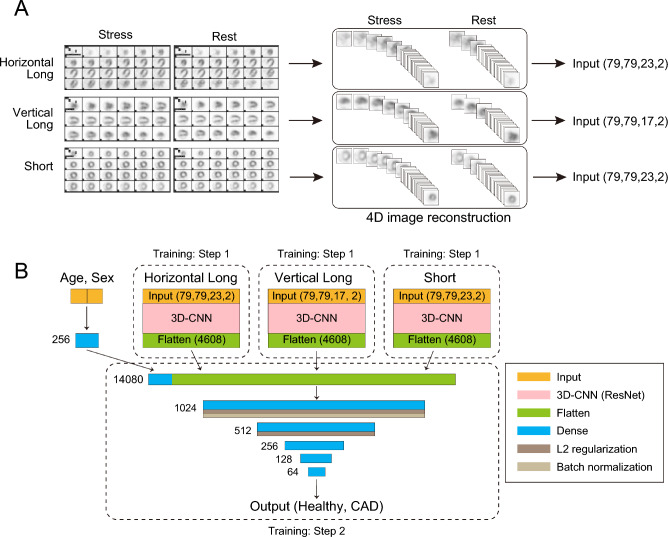
Preparing the dataset of MPI SPECT images for AI training. (**A**) MPI SPECT image-processing protocol for training. Six continuous cross-sections of grey scale processed SPECT images were used for training: three axes (horizontal long axis, vertical long axis, and short axis) and two conditions (rest and stress) for each axis. Each continuous cross-section was stacked in three dimensions, the resting and stress images of the same cross-section were combined, and 4D-data for each axis. (**B**) Structure of the AI model. We constructed a 3D-CNN based on a deep residual network, and 4D-data were input to the 3D-CNN as two-channel 3D data. The 3D-CNN processed each data from three axes: the horizontal long axis, vertical long axis, and short axis. In training step 1, we trained the model using only a single-axis dataset (either horizontal long, vertical long, or short), without age and sex information. In training step 2, the top layers before the dense layers of the step 1 models were transferred to the model with three branches, including sex and gender information. An answer was output based on whether the image indicated healthy or coronary artery disease. MPI SPECT, single-photon emission computed tomography for myocardial perfusion imaging; CNN, convolutional neural network.

To address these issues in the present study, we first created four-dimensional (4D) datasets, which contained both the resting and stress images of the three-dimensional (3D) stacked cross-section (Fig. 1A). Then, we constructed a 3D convolutional neural network (CNN) based on a deep residual network^29–32^, and the 4D-data were inputted to the 3D-CNN as two-channel 3D data. The 3D-CNN processed the data from three axes: the horizontal long, vertical long, and short axes. The output of the three 3D-CNNs was combined with the age and sex information, and the probability of the CAD images was calculated (Fig. 1B). Additionally, we developed an algorithm capable of focal diagnosis of CAD lesions^33–36^. We used two methods for focal diagnosis, Grad-CAM and unsupervised learning. Grad-CAM visualizes parts of an image that are crucial for AI-based diagnosis^37^; focal diagnosis can be possible using Grad-CAM because a trained-AI may recognize areas of decreased blood flow. Furthermore, a more precise focal diagnosis may be achieved by using unsupervised learning, such as principal component analysis (PCA)^38,39^, because PCA may present greater accuracy in grouping from a trained model; thus, abnormal CAD images can be classified according to CAD lesions. Finally, we compared the diagnostic performance of our model with that of cardiologists and demonstrated the usefulness of the AI-based system.

## Results

### The performance of classification by our system

**Figure 2:**
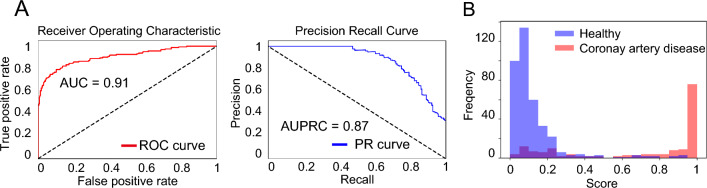
Performance of AI model in test dataset. (**A**) AUC of the ROC curve and AUPRC in the test dataset. (**B**) Histogram showing the frequency of the final output in each image. Blue and red bars show the output from the images labeled as healthy and CAD, respectively. AUC, area under the curve; ROC, receiver operating characteristic; AUPRC, area under the curve of the precision recall curve; CAD, coronary artery disease.

**Figure 3:**
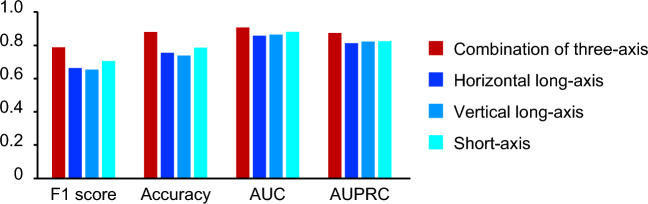
Comparison of three-axis and single-axis model. Indexes: F1 score, accuracy, AUC, AUPRC evaluated by three-axis AI model (Final model) or three kind of single-axis AI model (horizontal long, vertical long, and short axis) in the test dataset. AUC, area under the curve; ROC, receiver operating characteristic; AUPRC, area under the curve of the precision recall curve.

Table 1 shows the baseline characteristics of the patients. The trained deep neural network was able to classify MPI SPECT images as healthy or CAD with high accuracy in the test data that were not used for training. The area under the curve (AUC) of the receiver operating characteristic (ROC) curve was 0.91, and the area under the curve of the precision recall curve (AUPRC) was 0.87 (Fig. 2A, Table S1). The histogram in Fig. 2B shows that the probability of CAD images mainly ranges from 90 to 100%, while that of non-CAD images ranges from 0 to 10%. In total, 14.2% of images included noises or artifacts in our datasets. The performance of the non-artifact images (AUC: 0.93, AUPRC: 0.90) was much higher than that of the artifact images (AUC: 0.74, AUPRC: 0.78) (Fig. S2A,B, Table S2). Next, we compared the combination of three axis, horizontal long, vertical long, and short axes. The AUC and AUPRC were 0.91 and 0.87 for the three-axis model, 0.86 and 0.81 for the horizontal-long-axis model, 0.87 and 0.82 for the vertical-long-axis model, and 0.88 and 0.83 for the short-axis model, respectively (Fig. 3, Table S3). In addition, the performance of model using both stress and rest images was higher than that using only stress images (Table S4).

**Table 1:** Patient demographic information.

	Healthy	CAD
Number of studies	3801	1642
Age (year ± SD)	70.1 ± 11.25	70.36 ± 9.82
Age groups		
≦20, n (%)	4 (0.1)	1 (0.1)
20–40, (%)	53 (1.4)	7 (0.4)
40–60, (%)	529 (13.9)	206 (12.5)
60–80, (%)	2468 (64.9)	1129 (68.8)
> 80, (%)	747 (19.7)	299 (18.2)
Sex groups		
Male, n (%)	2553 (67.2)	1396 (85.0)
Female, n (%)	1248 (32.8)	246 (15.0)

### Detection of diseased area using trained model

**Figure 4:**
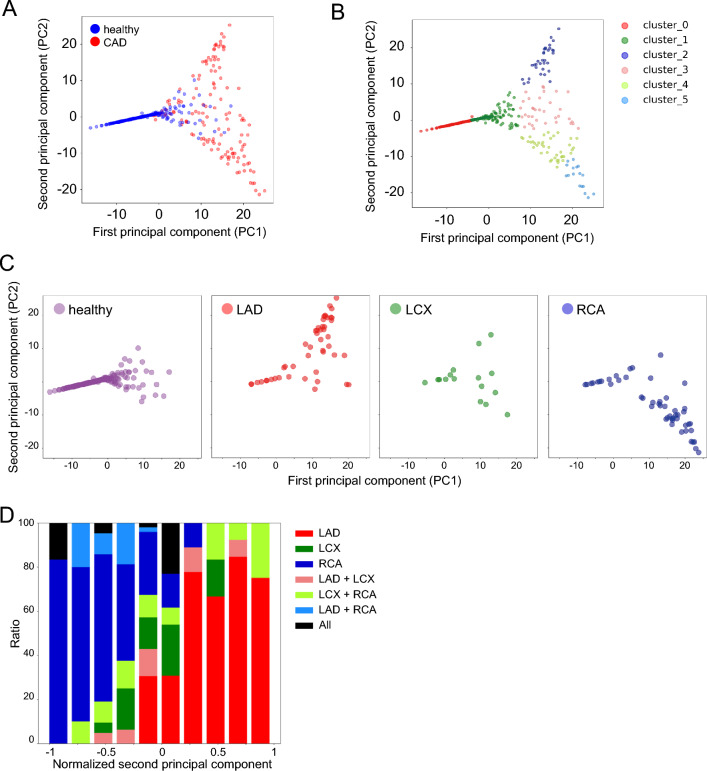
Detection of diseased area using principal component analysis (PCA). (**A**) PCA of the output of the 512-dimensional second dense layer after concatenation. The scatter plot shows the PC-1 and PC-2 value. Blue point: healthy; red point: CAD. (**B**) k-means +  + clustering of PCA plots (*k* = 6). (**C**) PCA plot of the image with a lesion in the LAD, LCX, and RCA alone. (**D**) Ratio graph of min–max normalized PC-2 value in each coronary artery lesion. PCA, principal component analysis; PC, principal component; LAD, left anterior descending artery; LCX, left circumflex artery; RCA, right coronary artery.

**Figure 5:**
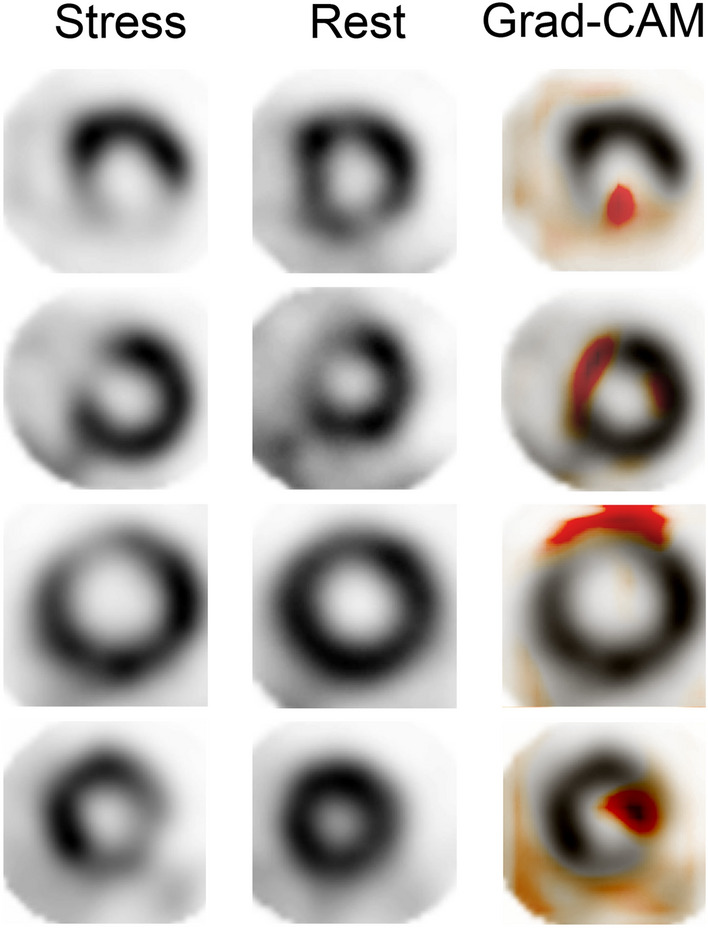
Visualization of diseased area using Grad-CAM. Representative Grad-CAM images showing lesions in MPI SPECT images of coronary artery disease. Short axis image in the stress condition (left), rest condition (center), and Grad-CAM merged image in the stress condition (right). MPI SPECT, single-photon emission computed tomography for myocardial perfusion imaging.

Next, we examined whether the trained model could visualize which part of the coronary blood flow reduced. First, we utilized a feature map extracted from the second dense layer of the model and performed principal component analysis (PCA). PCA showed that images of CAD were heterogeneous, and that they could be detected as four clusters (Clusters 2, 3, 4, and 5) by k-means clustering methods (Fig. 4A,B, Fig. S3A). In contrast, the images labeled as healthy were relatively uniform and formed a compact cluster (Clusters 0 and 1) (Fig. 4A). We annotated CAD images based on diagnostic reports in which major coronary branches were suspected to be diseased lesions (Fig. S3B). The lesions of three main branches, including left anterior descending artery (LAD), left circumflex artery (LCX), and right coronary artery (RCA), were identified as different clusters based on k-means clustering (Fig. 4C). In particular, images of LAD lesions were classified as cluster 2 or 3, whereas RCA lesions were almost classified as cluster 4 or 5 (Fig. 4B,C, Fig. S3C). The LAD and RCA images were in opposite locations in the second principal component (PC-2) (Fig. 4C). Hence, the axis of PC-2 represented the site of the CAD lesion in the short axis of the heart, while the axis of the first principal component (PC-1) represented whether the images indicated CAD or not (Fig. 4A,C). Indeed, the ratio graph showed that the location of the disease lesion changed continuously, depending on the value of normalized PC-2 (Fig. 4D). Additionally, we attempted to visualize the defect area of blood flow using Grad-CAM methods^30^. Grad-CAM can be used to visualize parts of an image that are important for AI judgment. We succeeded in visualizing the lesions where the coronary blood flow deteriorated on MPI images (Fig. 5).

### Comparison with cardiologists not specialized in nuclear medicine

**Figure 6:**
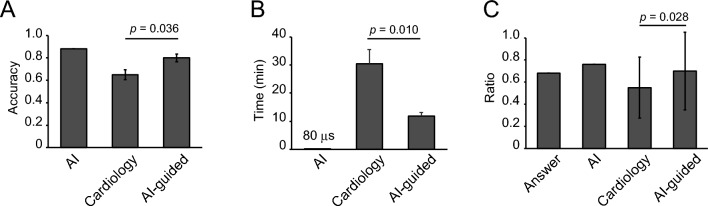
Comparison of diagnosis between AI and cardiologists. (**A**) Accuracy of AI diagnosis, cardiologist diagnosis, and cardiologist diagnosis with reference to AI diagnosis (AI-guided) in 50 randomly selected MPI SPECT images. (**B**) Time spent diagnosing 50 MPI SPECT images. (**C**) Ratio of MPI SPECT images diagnosed as CAD to total 50 images. MPI SPECT; single-photon emission computed tomography for myocardial perfusion imaging.

The performance of the AI-based diagnosis system was compared with the diagnostic accuracy of cardiologists who were not specialized in nuclear medicine. We determined three cutlines for the AI model and provided the reliability for the model based on the probability of CAD, which was provided as an output by the deep neural network: over 90% and under 10% probability was a highly reliable zone for the AI model (62% of the total images, accuracy: 0.94, PPV: 0.97, NPV: 0.94); from 80 to 90% and from 10 to 20% were a moderately reliable zone for AI-judge (22% of the total images, accuracy: 0.88, PPV: 0.86, NPV: 0.88); and from 20 to 80% was a low reliability zone for the AI model (16% of the total images, accuracy: 0.62, PPV: 0.55, NPV: 0.7) (Fig. S4A,B, Table S5). The accuracy was 0.88 in the AI model, 0.80 in the AI-guided approach, and 0.65 in without-AI (Fig. 6A). The time required for diagnosis was shorter for AI-guided diagnosis (11.8 min) than for that without AI guidance (30.5 min). Notably, the AI model took only 80 µs for diagnosis (Fig. 6B). We calculated the ratio of MPI SPECT images diagnosed as CAD to total images, and found that the ratio was higher in cardiologists without AI guidance (0.45) than in the AI-guided diagnosis (0.30) (Fig. 6C), indicating that cardiologists tend to over diagnose artifacts as CAD, and that AI can reduce bias in diagnosis.

## Discussion

In this study, we succeeded in constructing an automated AI-based system for MPI SPECT images to predict CAD with high performance (AUC: 0.91, AUPRC: 0.87) (Fig. 2A), and, as shown by the histogram of output probability, the model could clearly separate healthy and CAD images (Fig. 2B). In addition to the improvement of diagnostic accuracy, our AI model can also support clinicians by reducing interpretation time. Our deep learning-based diagnostic support system offers significant clinical benefits by enhancing diagnostic accuracy and efficiency for cardiologists.

Recently, several studies have shown the usefulness of machine learning technology^19–21^ and deep learning-based systems using large datasets of MPI SPECT images^24–28^ for cardiovascular disease prediction. Compared to those of previous studies^24–28^, we successfully constructed the AI model with high performance. The high performance of our AI system stemmed from its utilization of three axes within four-dimensional datasets, coupled with comprehensive patient biographical information. In previous reports, 2D images such as polar maps or stress-only images were often used^24–28^. However, these quantitative methods compress original data into 2D representations to facilitate the evaluation of perfusion defects. While useful, this compression can lead to a decrease in detailed spatial information inherent in the original data. With the improvement in GPU performance, it has become possible to directly input 3D images into deep-learning models. Our approach indeed achieved higher classification performance, suggesting that retaining the 3D data provides a more comprehensive analysis of myocardial perfusion, leading to better diagnostic outcomes. We believe that we have successfully extracted the maximum features of the MPI SPECT images by inputting 3D images and two separate channels for the resting and stress states. Moreover, by incorporating three axes of multi-section tomographic images and clinical information, such as age and sex, we have successfully constructed a high-performance model. Interestingly, the short-axis model had the highest performance among the three single-axis models (Fig. 3). This corresponds to the interpretation of nuclear medicine specialists, which suggests that the performance of the model we constructed is comparable to that of the actual interpretation method by a specialist. To achieve high accuracy in classification using deep learning, a large number of cases are required. Using radiology reports as the teacher data in our study, we were able to incorporate all images of MPI SPECT (7128 images), which were obtained from Keio University Hospital between May, 2012 and March, 2021, into the study. We believe that this contributed to the improvement of the accuracy and generalization of the system.

Another novel aspect of our study was that we proposed two complementary methods for accurately predicting the spatial localization of coronary artery lesions. In general, it is difficult to obtain physiological information beyond that of what is labeled in supervised learning. However, in this study, we succeeded in extracting information regarding coronary artery lesions without teacher data. We visualized the diseased area using two methods: unsupervised learning and Grad-CAM. Interestingly, PCA showed that we could identify which coronary arteries were diseased, even though that information was not included in the training data label. These data indicate that the training model, which only performs binary classification, retains the information of the entire MPI SPECT image, therefore suggesting that not only classification can be performed, but also a more detailed diagnosis. Moreover, Grad-CAM, which can visualize important regions for decision making by AI, also showed that areas of reduced blood flow, which indicated coronary artery lesions, were important for classification. This suggests that the trained AI could correctly extract the features of the image. Our method, which applies and links trained AI to a more detailed diagnosis, can be an important representative method for utilizing AI for medical images in the future.

We compared the performance of AI with that of cardiologists who were not specialized in cardiac nuclear medicine and revealed the usefulness of the AI model. In actual clinical practice, diagnosis is not solely based on AI decisions, but is instead a combined result of clinician experience and AI assistance. Moreover, the AI guide shortened the time required for diagnosis by the cardiologists without reducing the accuracy. An MPI image sheet includes approximately 140 images for a patient, and it can be a mental and physical burden for attending physicians. Additionally, it has been reported that faster image reading rates are associated with diagnostic errors in radiology. In particular, a rate faster than six cases per hour increases diagnostic errors significantly^40^. Given the shortage of radiologists, our image-reading system, which reduces physician burden and shortens image interpretation time, can be a powerful tool in real-world clinical settings.

Our dataset was collected from a single center, Keio University; thus, it is unclear whether our system can be applied to other facilities. However, we included all images that were available at Keio University for 9 years, thereby contributing to the high accuracy and robustness of our system. Moreover, we propose the fundamental strategy for the diagnosis of CAD using MPI SPECT. Our system can be applied to other facilities by performing additional learning and model training on their datasets.

In conclusion, we successfully constructed an AI-based automated system for diagnosing CAD using MPI SPECT images. This system led to make more effective and accurate diagnoses by clinicians.

## Methods

### Study population

This retrospective study included a total of 7128 patients who underwent stress/rest MPI SPECT between May 2012 and March 2021 at Keio Hospital. Demographic data, including age and sex, were retrieved from our electronic medical records.

### Image labeling and dataset division

We labeled each MPI image by referring to the diagnostic reports of MPI interpretation. The images were visually interpreted by specialists of cardiac nuclear medicine after incorporating all available data, including stress and rest perfusion imaging and clinical information. The datasets included artifacts from tissue attenuation of radioactivity, small defects, or defects representing other conditions (e.g., body motion during imaging causing a significant loss of image quality), which were considered suggestive of no flow-limiting CAD and labeled negative for CAD. Among the 7128 images, after excluding 1685 images that were unclassifiable through data extraction, we utilized 5443 images, including 3801 healthy images (definite: 3385, artifact: 416) and 1642 CAD images (definite: 1284, CAD: 358) for machine learning.

Next, we randomly extracted 10% of the available data for the test dataset; 10% of the remaining data were used for validation, and the remaining data were utilized for training. Separation was performed for each label. The training dataset included 4356 cases (healthy: 3041, CAD: 1,315), the validation dataset included 542 cases (healthy: 379, CAD: 163), and the test dataset included 545 cases (healthy: 381, CAD: 164) (Fig. S1). All programs were written in Python 3.

### Stress protocol

All patients underwent standard pharmacological or exercise stress Tl-201 MPI. Exercise stress tests were performed using a bicycle ergometer with multi-stage loading, starting at 25 W or 50 W and increasing by 25 W every 3 min. When the target heart rate or standard exercise endpoint was reached, 111 MBq of radioisotope Tl-201 was administered. Exercise at the same level was continued for another minute post-radiotracer injection. In the pharmacological stress test, dipyridamole at a dosage of 0.56 mg per kilogram body weight was administered slowly intravenously over 4 min, followed by a 2 min-later administration of 111 MBq of the radioisotope Tl-201.

### SPECT image acquisition and processing

Fifteen minutes and 3–4 h after injection of 111 MBq Tl-201, MPI SPECT images were obtained using a double-headed gamma camera (Discovery NM/CT 670, GE Healthcare, Milwaukee, WI, USA) equipped with a low-energy high-resolution collimator with the patient in the supine position. SPECT data were collected in 60 views in steps of 6°, with each detector rotating 180° (360° acquisition). The scan durations were 10, 12.5, and 15 min for patients weighing < 60 kg, 60–70 kg, and ≥ 70 kg, respectively. All images were processed with commercially available software on a GEniE-Xeleris workstation (GE Healthcare, Milwaukee, WI, USA). Data were reconstructed using the ordered subset expectation maximization method with a Butterworth filter (order, 10; cut-off frequency, 0.30–0.34 cycles/cm). The matrix size for the data acquisition and image reconstruction was 3.4 mm (128 × 128).

### Preparation of input data for the model

There were six types of SPECT images for every patient, comprising images of three axes (horizontal long, vertical long, short) and two conditions (stress, rest) for each axis. We utilized all six types of SPECT images, including both rest and stress SPECT images for each axis, in our study. The images of the horizontal long and short axes had 23 slices, and those of the vertical long axis had 17 slices. The image size was 1200 × 1640 or 1080 × 1640 pixels depending on the imaging equipment. To generate a dataset for machine learning, we cropped the SPECT images of each slice from the image sheets. The acquired images were converted to grayscale (0–255). Each slice was aligned at the center and cropped with a small image margin. The coordinates for cropping were identified using ImageJ software. The size of each image was 79 × 79 pixels, which was determined to maximize the original information value of the images and minimize the calculation cost for training. The cropped images were then converted into a NumPy array. SPECT images corresponding to each slice were concatenated to generate cubic images (79 × 79 × 23 or 79 × 79 × 17 voxels). Next, we concatenated two cubic images of the stress/rest conditions to create cubic images with two channels (79 × 79 × 23 × 2 or 79 × 79 × 17 × 2 voxels). The last channel represents the stress and rest conditions.

### Training of deep CNN model

We designed a 3D-CNN for training, which used three branches with the input of three axes that were taken simultaneously (branch 1: horizontal long axis; branch 2: vertical long axis; branch 3: short axis). The proposed approach uses deep features of ResNet-34 composed of 3D-CNN. ResNet-34 is a 34-layer CNN that uses skip connections and shortcuts to jump over some layers, enabling the avoidance of vanishing gradient and degradation problems even with deeper models. Each convolutional layer was followed by rectified linear units (ReLU) for activation and batch normalization, and the final layer of ResNet 34 was fed to a global average pooling layer.

The outputs of all branches were concatenated with 256-hidden-units-layer of patient demographic data, including age and sex data. These outputs were then connected with six dense layers of 1024, 512, 256, 128, 64, and 1 unit. The first two dense layers applied the L2 regularization penalty (L2 = 0.01) to the loss function, the batch normalization layer was applied after the first dense layer to avoid overfitting, and the last layer applied a sigmoid function to calculate the probability of the classification. We utilized stochastic gradient descent as a loss function. The model was trained to minimize the loss function.

The learning procedure was divided into two steps. In the training step 1, we trained the model using only a single-axis dataset (horizontal long, vertical long, and short axes), without age and sex information. In the training step 2, the top layers before the dense layers of step 1 models were transferred to the model with three branches, including sex and gender information. The weights of the transferred top layers were fixed, and only those of the dense layers were updated. In the first and second steps, the initial learning rates were 0.1 and 0.03, respectively. Both were reduced by a tenth when the value of the validation loss function was not improved for 10 epochs during training, and the training was stopped when the value was not ameliorated for 20 epochs. The reduction in the learning rate and the early stopping method prevented overfitting. The input images were normalized as follows:

1 $$ {\text{Y}} = \left( {\left( {{\text{X}}/{255}} \right){-}0.{5}} \right) \times {2} $$

(Y: value of normalized images; X: value of original images).

### Data augmentation

For data augmentation, we simultaneously applied a slight rotation of the original images by a maximum of five degrees, a change in brightness by a maximum of 0.1 times for each pixel, zooming 0.8–1.0 times, and shifting, in which each slice randomly moves the margin space caused by zooming. The augmented images were concatenated to generate new images. In this way, data augmentation enables the dataset to be increased without degrading the information and modifying the imbalance between classes, which helps reduce overfitting when training a machine learning model. Thus, the training performed better with an augmented balanced dataset than with an original imbalanced dataset. The CAD and non-CAD datasets were augmented to 12,000 images each. The same procedure was used for images of each axis.

### Evaluation of model performance

We obtained 545 images in the test dataset, including 381 healthy images (definite: 339, artifact: 42) and 164 CAD images (definite: 128, artifact: 36). The probability value of the test dataset was calculated to evaluate the performance of the trained model. The test dataset was not utilized for the training; thus, the predicted result of the test dataset reflects the model’s versatility and performance. Our dataset included MPI images with artifacts, and it is evident that artifact images are far more difficult to correctly interpret than non-artifact images, even for cardiologists and radiologists. To confirm the difference in prediction accuracy between artifact and non-artifact images, we separated the test data into artifact and non-artifact datasets and calculated the evaluation score. Further, we trained models using only a single axis without age and sex data to clarify which axis is prone to reflect the CAD features the most and to clarify the difference in the predictive performance between the three-branch model and single-axis model.

### Unsupervised learning for prediction of coronary artery lesion

To examine the internal features learned by the CNN, we visualized the features using PCA^38^. We used PC-1 and PC-2 to generate scatter plots. Each point represents an MPI image in the test dataset projected into two dimensions from the 512-dimensional output of the second layer after the concatenation layer of the three-axis CNN. To determine the PC-2 value, we performed cluster analysis using the PC-1 and PC-2 values with k-means++ as a clustering analysis method^39,41,42^. We separated the test data into six clusters and confirmed the frequency of coronary artery branches that caused CAD in each cluster. The involved branches were identified by referring to diagnostic reports of the MPI images from radiologists.

### Grad CAM

We used the Grad-CAM method to visually present the predictions from a deep neural network^37^. Grad-CAM, which stands for gradient-weighted class activation mapping, is a method used to produce a coarse localization map highlighting important regions in the image for predicting the concept. Grad-CAM calculates the gradients of a target flowing through the network and determines the importance of each neuron in a network.

### Cardiologist survey

To compare the diagnostic performance of our CNN model with that of cardiologists, we designed a series of surveys for four independent cardiologists. Fifty cases were randomly selected and shown to the cardiologists, who were blinding to the model outcomes. They were then asked to determine the cases diagnosed with CAD by radiologists. We presented grayscale and colored images that can draw a sharp contrast in nuclear attenuation. In addition to the images, we revealed patient characteristics, including age and sex. After the first interpretation, we showed the same dataset with the possibility of CAD, which was predicted by our model, and asked them to diagnose the MPIs again. Before the second interpretation, we explained the test performance of our model, including the accuracy, positive predictive rate, and negative predictive rate. Before completing the second interpretation, we did not present the results of the first interpretation. Finally, the results and time taken for interpretation were compared using two-tailed paired *t*-tests.

### GPU server and analysis environment

We used the GPGPU server, which has two CPUs: Xeon Silver 4210R 2.40 GHz, 768 GB CPU memory, and two GPUs, Quadro RTX8000 48 GB GDDR6 (NVIDIA, Santa Clara, CA, USA). All scripts were programmed on the Nvidia-Docker system with Ubuntu 20.04, CUDA 11.2, cuDNN 8.1, Anaconda 4.11.0, Python 3.7.7, and Tensorflow 2.4.0.

### Statistical analysis

Network performance was evaluated by the accuracy, precision, recall, F1 score, AUC of the ROC, and AUPRC. We determined the threshold value that was utilized for assigning predicted labels by maximizing the F1 score. All possible values equal to or greater than the threshold were mapped to the CAD class, and all other values were mapped to the healthy class. The threshold was set to 0.30. Accuracy was the ratio of correct predictions to all the predictions, while precision was the positive predictive rate of predictions, defined as the number of true positives divided by the number of true positives plus false negatives. Recall was the sensitivity of the prediction, which was defined as the number of true positives divided by the number of true positives plus false positives.

2 $$Accuracy=\frac{TP+TN}{TP+FP+TN+ FP},$$

3 $$Precision = \frac{TP}{TP+FP},$$

4 $$Recall=\frac{TP}{TP+FN}$$

The F1 score is the harmonic mean of the precision and recall:

5 $$F1 score = \frac{2 Recall \times Precision}{Recall+Precision}$$

The ROC curve is a plot of the true positive rate against the false positive rate for all the possible thresholds. The AUPRC is a plot of the precision value against the recall value.

### Approval for human experiments

The experiments of the study were approved by the Keio University School of Medicine Ethics Committee (No. 20170187). Informed consent was obtained from all patients in the form of opt-out for the retrospective analysis of patient images. Patients were provided with information about the purpose and content of the study on the website and were given the option to refuse participation if they did not wish to be included. The experiments were performed in accordance with the declaration of Helsinki and with all guidelines set forth by the approving Institutional Ethics Committee. The all data were completely analyzed anonymously.

### Supplementary Information

Supplementary Information.

## Data Availability

All data generated or analyzed during this study are included in this published article and its supplementary information files.
