# Supplementary material for: A deep learning-based automated diagnosis system for SPECT myocardial perfusion imaging
Source: Sci Rep. 2024 Jun 12;14:13583. doi: 10.1038/s41598-024-64445-2 (PMC11169468; doi:10.1038/s41598-024-64445-2)
Supplement: Supplementary file 1 — Supplementary Information. [file 41598_2024_64445_MOESM1_ESM.docx]

**Supplemental Figures and Tables**

**Figure S1. Study population**

Summary of the data used in this study. All MPI SPECT images (7128 images) obtained at Keio Hospital from May, 2012 to March, 2021 were considered in this study.

**Figure S2. Changes in parameters with and without artifacts**

**(A, B)** AUC of the ROC curve (left graph), AUPRC (middle graph), and histogram of the AI output in dataset **(A)** which does not include artifacts or noise, or (**B)** which includes artifacts or noise.

**Figure S3. Results of unsupervised learning**

**(A)** Percentage bar graph showing the frequency of normal and abnormal values in each K-means + + clustering cluster. (**B)** Principal component analysis (PCA) of the output of the 512-dimensional second dense layer after concatenation. The scatter plot shows the first principal component (PC-1) and the second principal component (PC-2) value. The color of each plot represents the diseased lesion. (**C)** Percentage bar graph showing the frequency of clusters 2–5 for each disease lesion.

**Figure S4: Changes in parameters depending on the reliability of AI prediction**

**(A)** Histogram showing the frequency of the final output in each image. The test data divided into high reliable zone (probability value <0.1 or >0.9), moderate reliability zone (probability value 0.2~0.3 or 0.8~0.9), and low reliability zone (probability value 0.2~0.8). (**B)** Accuracy, PPV, and NPV of predicted values in high, moderate, and low reliability zones. PPV, positive predictive value; NPV, negative predictive value.

**Table S1**. Quantitative results of the optimal AI model in the test dataset

**Table S2**. Changes in evaluation parameters with and without artifacts

**Table S3**. Quantitative results were evaluated using four different models: three-axis AI model (final model) and three kinds of single-axis AI model (horizontal long, vertical long, and short axes) in the test dataset.

**Table S4**. Changes in evaluation parameters using both rest and stress images and using only stress images.

**Table S5**. Quantitative results in a highly reliable, moderately reliable, and low-reliability zone.
